# Non-Smoking Tobacco Affects Endothelial Function in Healthy Men in One of the Largest Health Studies Ever Performed; The Nord-Trøndelag Health Study in Norway; HUNT3

**DOI:** 10.1371/journal.pone.0160205

**Published:** 2016-08-04

**Authors:** Eli-Anne Skaug, Bjarne Nes, Stian Thoresen Aspenes, Øyvind Ellingsen

**Affiliations:** 1 K. G. Jebsen Center of Exercise in Medicine, Department of Circulation and Medical Imaging, Norwegian University of Science and Technology, Trondheim, Norway; 2 Department of Cardiology, St. Olav’s Hospital, Trondheim, Norway; Hunter College, UNITED STATES

## Abstract

**Background:**

Oral tobacco (snuff) is taking a large market share in Scandinavia, especially with young users. However, long-term health effects are unknown. Small studies show association between snuff and reduced endothelial function, representing an early stage of vascular injury that often precedes manifest cardiovascular disease by several years. We therefore determined the associations between snuff and endothelial function in a large sample of healthy Norwegian men.

**Methods and Design:**

In the Fitness substudy of the Nord-Trøndelag Health Study (HUNT3), endothelial function was measured by flow-mediated dilation (FMD). Aerobic fitness was measured by peak oxygen uptake (VO_2peak_). A cross-sectional design including 1 592 self-reported healthy men compared these observations with records of present tobacco use, standard cardiovascular risk factors, and socioeconomic status, using general linear models.

**Results:**

FMD was lower in snuff users (FMD: 4.12%, 3.63, 4.61) compared to non-users (FMD: 4.52%, 4.27, 4.78) after adjustment for age (difference: -0.57%, -1.12, -0.01). After further adjustment for potential confounders, FMD still tended to be lower in snuff users than in non-users (difference: -0.53%, -1.09, 0.02). This difference was even more pronounced in the inactive snuff users (-0.83%, -1.59, -0.06) and in the low fit snuff users (-0.74%, CI -0.55, 0.079).

**Conclusions:**

Oral tobacco is associated with a tendency towards reduced endothelial function, indicating vascular changes that precede cardiovascular disease. The strongest associations were found in men with low physical activity or reduced aerobic fitness.

## Introduction

Smoking tobacco is a core risk factor for cardiovascular disease (CVD) [1–4]. In the last 30 years the consumption has decreased significantly in Norway [5,6]. However, the use of oral tobacco (“snuff”) has been rising, especially in younger age groups [7]. This observation has prompted a debate about long-term consequences and whether it is safe to recommend snuff as an alternative to smoking tobacco. Most studies reporting associations between tobacco use and future cardiovascular disease have focused on long-term endpoints like heart failure, ischemic heart disease, stroke, and mortality, which are less prevalent at younger age groups and requires several years of follow-up [8].

Endothelial dysfunction is an early marker of atherosclerosis associated with conventional risk factors for coronary heart disease including cigarette smoking. It is also a typical finding in peripheral artery disease [9–12]. Therefore, assessment of endothelial function measured as flow mediated dilatation (FMD) of the brachial artery may be a useful method to detect persons with increased cardiovascular risk [13–16]. FMD is positively associated with physical activity [17,18] and negatively associated with age [19]. Vascular endothelial cells are adversely affected by smoking tobacco [20,21], and some studies have indicated impaired endothelial function after snuff use [22,23]. However, these studies were either performed in oral mucosa [24] or with a low number of as few as 20 study-participants [24,25]. There is some evidence that snuff aggravates the risk of cancer [26], but somewhat equivocal findings whether it induces atherosclerosis and enhances the risk for cardiovascular disease [27–31]. An accumulating number of studies show associations between use of oral tobacco products and the risk of fatal myocardial infarction and stroke [32–35]. Metabolic syndrome has been associated with use of snuff [36,37], and in Norway pregnant women are recommended to refrain from using snuff because of higher risk for preeclampsia, which is associated with endothelial dysfunction and preterm birth [38,39]. Given the incomplete knowledge of long-term effects on endothelial function, we conducted a population-based study to determine the association between the use of snuff and endothelial function measured by FMD.

## Study Population

### Inclusion and Exclusion

In Nord-Trøndelag County, all inhabitants from 20 years of age and up (n = 94 194) were invited to participate in the third wave of the Nord-Trøndelag Health Study (HUNT3), which was carried out between October 2006 and June 2008 [40]. Among the 50 821 who attended (54%), 30 588 were defined as healthy, based on a self-administered questionnaire (Q1[41]). From those defined as healthy, a random sample of 12 609 were invited to participate in the HUNT Fitness Study, a sub-study conducted in four selected municipalities within the county. Inclusion criteria were participation in HUNT3 and written informed consent. Besides FMD, the Fitness Study included peak oxygen uptake (VO_2peak_) measured by ergospirometry, a questionnaire assessing physical activity, and a clinical examination[42]. FMD from the Fitness Study was combined with data from the main HUNT Study, including questionnaire information regarding present tobacco use [43] and physical activity [43], anthropometric variables, blood pressure and serum lipids, and with socioeconomic data from Statistics Norway. Participation was voluntary and 5 633 accepted the invitation for FMD testing.

Exclusion criteria were wheezing or dyspnea during the past 12 months, previous asthma, chronic obstructive pulmonary disease, sarcoidosis, cancer, cardiovascular disease, including cerebral and peripheral arterial disease, angina and previous myocardial infarction, contraindications towards physical activity, and the use of antihypertensiva or vasoactive medication known to influence endothelial function. Respondents with arrhythmias, including atrial fibrillation and ventricular bigeminy during testing were excluded. In total, 2 211 men and 2 528 women completed FMD measurements. Since only 6 women used snuff daily, further analyses were performed on men only. Health characteristics of these men is described in details in a previous study [44]. Participants with inconclusive answers regarding earlier use or non-use of any kind of tobacco were excluded from analysis. After excluding respondents with missing data 1 592 men remained.

### Characteristics of Background Population

Socio-economic status, disease epidemiology and risk factors of the study population have previously been described [6,7,15,19,45], and the respondents are representative of the general healthy Norwegian population [45,46]. In Norway 14% of men in the age group 16–74 years report daily smoking and 16% use snuff daily. The proportion of occasional snuff users amongst men is 6% [7]. There is little variation among snuff brands in Norway; all are produced in Sweden by a heating process [47], and the level of nitrosamine is reportedly 0.5–3.1 micrograms per kilogram [48].

## Methods

### Flow Mediated Dilatation

Flow-mediated dilation (FMD) of the left brachial artery was measured with ultrasound and Doppler (Vivid I, GE Healthcare, USA) using a high-resolution 12 MHz array transducer (Vivid, GE Healthcare, USA). A blood pressure cuff was placed on the forearm [49,50] with the participants in supine position in a dark and quiet room with a stable temperature. Blood flow was recorded by pulsed Doppler velocity signals. The artery was imaged at baseline after ten minutes of supine rest, and 60 seconds after cuff deflation following arterial occlusion at 250 mm Hg for five minutes. The mean of three consecutive arterial measurements was recorded using 0.1-mm resolution calipers. Diameter was measured from intima to intima at the peak of the R-wave to avoid confounding by cyclic changes in arterial dimension. FMD was defined as percent change in vessel diameter, calculated as [(post occlusion diameter–baseline diameter) / baseline diameter]. Inter-observer analysis with Pitman's Test of difference in variance ranged from r = 0.008 (n = 81, p = 0.942) to: r = -0.846 (n = 82, p = 0.000) [45]. Endothelial dysfunction (ED) was defined as FMD ≤ 0%. All participants received written information beforehand and were asked to fast and refrain from coffee and tobacco the last four hours before testing. Testing was part of the present cross-sectional health survey and there exist no measurements on FMD prior to recruitment in study.

### Blood Pressure, Anthropometrics and Blood Analyses

Resting heart rate was recorded as the lowest heart rate during supine rest for 10 minutes. Blood pressure was measured (Critikon Dinamap 845XT, GE Medical Systems, USA), sitting according to guidelines [51]. Combined scales (Model DS-102, Artic Heating AS, and Norway) measured height and weight to the nearest cm and kilogram, and body mass index (BMI) was calculated. Waist circumference was measured at the umbilical level. Blood samples were drawn right after blood pressure measurements, and then centrifuged and refrigerated before transportation to the analysis laboratory on the same day. Serum analyses were performed in fresh samples. All analyses were performed by photometric methods on an Architect ci8200 (Abbott Laboratories, IL, USA). The coefficients of variation at the laboratory were 1.4–1.7% for glucose, 1.1–1.3% for cholesterol and 1.0–1.7% for high-density lipoprotein cholesterol. Peak oxygen uptake (VO_2peak_) was measured by ergospirometry during treadmill running or walking until exhaustion [52]. Physical activity was evaluated by a previously published index score and categorized according to current recommendations [53].

### Confounders

Regression models adjusted for potential confounders; such as age, education, income, physical activity, and systolic blood pressure. Previous studies have shown that physical activity has an effect on endothelial function (FMD) [54,55]. In Norway, snuff use is more prevalent in young men than in other groups of the population [7]. There is an inverse association between FMD and age [19]. Population studies have shown that low-income and low-educated participants have higher risk of cardiovascular disease [56] [44,57,58] as well as higher prevalence of other outcomes [59]. Systolic blood pressure is related to cardiovascular outcome and risks of cardiovascular disease [60]. In a previous study of the HUNT-population, we found an inverse association between systolic blood pressure (SBP) and endothelial function measured by FMD [61]. Further analysis revealed that the effect from tobacco on FMD was not associated with SBP (data not shown), and SBP was therefore not included in final models.

There were no differences in BMI between the tobacco-groups and non-user group (Table 1), and there was no association in our study population between FMD and BMI, waistline, or waist-hip-ratio. This has been described in our previous study [61]. These variables were therefore not included as confounders in the final models.

**Table 1:** Anthropometric data, risk factors and present tobacco use of study population.

	Non-user		Snuff, no smoke		Smoke, no snuff		Smoke and snuff	
N	886		238		447		21	
	Mean	95% CI	Mean	95% CI	Mean	95% CI	Mean	95% CI
**Age, years**	47.4	46.8–47.9	42.8	42.0–43.7	47.4	46.7–48.1	47.4	44.0–50.8
**Systolic BP, mmHg**	131	131–132	131	130–132	132	131–133	134	129.7–137.3
**Diastolic BP, mmHg**	76	75–76	76	75–77	76	76–77	77	75–80
**Waist circumference, cm**	95.0	94.6–95.4	95.0	94.3–95.7	95.1	94.6–95.6	96.6	94.3–98.9
**Body mass index**	26.4	26.2–26.6	26.7	26.3–27.1	26.4	26.0–26.7	26.3	24.8–27.9
**Non-fasting glucose, mmol/L**^**-1**^	5.48	5.42–5.55	5.35	5.27–5.42	5.54	5.46–5.62	5.62	5.32–5.93
**HDL-Cholesterol, mmol/L**^**-1**^	1.21	1.20–1.22	1.23	1.21–1.25	1.19	1.18–1.21	1.17	1.09–1.24
**Total-Cholesterol, mmol/L**^**-1**^	5.38	5.34–5.42	5.35	5.28–5.43	5.50	5.44–5.55	5.54	5.30–5.78
**Triglycerides, mmol/L**^**-1**^	1.75	1.71–1.80	1.78	1.69–1.86	1.92	1.85–1.98	2.05	1.75–2.35
**VO**_**2peak,**_ **mL·kg**^**-1**^**·min**^**-1**^	46.0	45.41–46.6	47.3	46.2–48.5	42.9	42.1–43.7	40.8	37.3–44.3
**Physical Activity Index**	3.4	3.2–3.6	3.5	3.1–4.0	2.5	2.3–2.8	2.0	0.8–3.1
**Metabolic Syndrome (%)**	17.8		14.9		21.9		25.4	
**Education, years**	13.7	13.6–13.8	13.6	13.4–13.8	12.6	12.5–12.8	12.3	11.7–12.9
**Income, 1000 NOK**	447	437–457	436	420–453	396	386–406	386	347–424

BP: blood pressure; HDL: high density lipoprotein, VO2peak: peak oxygen uptake; Income: average income 2007–2009

### Approvals

The Regional Committee for Medical and Health Research Ethics, the Norwegian Data Inspectorate and the Ministry of Health and Care Services, and Data Access Committee for HUNT Research Centre (DAC) approved of the HUNT3 study and the Fitness study. The Study Protocols conformed to the Helsinki declaration. Written informed consent was obtained from all participants.

### Restrictions in Use of HUNT-Data

Access to the requested HUNT data is given after the application is processed and a contract is signed. The contract gives the researcher(s) the right to investigate a specific topic for a limited time period and to publish a specified number of articles [62]. Participants in The HUNT Study have given a broad consent when entering into the study, agreeing to be included in studies unspecified at the time, under the condition that these are approved of by the Regional Ethics Committee. Each and every study conducted on HUNT data must therefore be approved of by a Norwegian Ethics Committee.

HUNT data are updated by quality assurance and with respect to participants withdrawing their consent for further analyses of data or use of biologic material. Old data files can therefore get outdated and may include participants who have expressed a wish to withdraw. Data cannot be stored in a public repository, as this would be a breach of the agreement with the participants in HUNT.

### Statistical Analysis

The study sample was divided into four groups based on self-reported present tobacco consumption; 1) no smoking or use of snuff at present, 2) use of snuff only, 3) smoking only, and 4) present smoking and use of snuff (Table 1). Descriptive data are given as means and 95% confidence intervals (CI) for continuous variables, and as percentage for dichotomous variables. The precision of the estimated means, mean differences and odds ratios are presented as 95% confidence intervals (in brackets). All data were examined for missing values and unlikely outliers. A general linear model with simple contrast and FMD as the dependent variable, and tobacco use as the categorical independent variables was applied to detect differences in endothelial function. Potential confounding variables were subsequently entered as covariates in different models. Model 1 was adjusted for age; model 2 was additionally adjusted for education and income, and model 3 also included the physical activity index [63]. Effect modification by age and physical activity was tested formally by inclusion of interaction terms. In subsequent analyses, tobacco status was combined with self-reported physical activity level or VO_2peak_ entered as categorical independent variable. The highest level of physical activity/VO_2peak_ with no tobacco use was set as reference category. High (“fit”) and low (“low fit”) VO_2peak_ were defined as above and below the average value for the respective 10-year age categories, respectively. Recommended physical activity (“active”) was set according to current national and international guidelines of >75 minutes of high intensity and/or >150 minutes of moderate intensity per week [64]. All statistical analyses were performed using SPSS software version18.

## Results

### Tobacco Use

In total, 447 (28.1%) men in the main analysis reported present smoking, 238 (14.9%) use of snuff, 21 (1.3%) smoking and snuff, and 886 (55.7%) no snuff or smoking (Table 1). Snuff users were younger than the average study population and younger than smokers (Table 1). Snuff users had higher education, income, physical activity level and VO2peak, as well as better cardiovascular risk profile (lower blood glucose, higher HDL-cholesterol and lower total cholesterol and triglycerides) compared to smokers (Table 1), but not compared to non-users (Table 1).

### Endothelial Function

After adjustment for potential confounders, flow mediated dilation (FMD) tended to be lower in snuff users (difference: -0.53%, -1.09, 0.02) than in the non-user group (Table 2). This difference was even more pronounced in the inactive snuff users (-0.83%, -1.59, -0.06, Table 3) and in the low fit snuff users (-0.74%, CI -0.55, 0.07, Table 4), respectively, compared to active or high fit participants that reported no tobacco use. Snuff users meeting current recommendations for physical activity level did not have lower FMD compared to the reference group (-0.29%, -1.25, 0.68), neither did the fit snuff users (-0.19%, -0.96, 0.57, Table 4). There was no difference between the reference group and the active smoking tobacco users (-0.41%, -1.35, 0.53) or inactive smoking tobacco users (-0.29%, -0.91, 0.33, Table 3). Formal tests of interaction were not significant for tobacco groups and physical activity recommendations (p = 0.56) or age (p = 0.93), respectively.

**Table 2:** Flow mediated dilation (%) according to present snuff-use and smoking.

		Unadjusted		Model 1		Model 2		Model 3	
	N	Mean	95% CI	Diff.	95% CI	Diff.	95% CI	Diff.	95% CI
**Non-user**	886	4.52	(4.27, 4.78)	Ref.	-	Ref.	-	Ref.	-
**Snuff, no smoke**	238	4.12	(3.63, 4.61)	-0.57	(-1.12, -0.01)	-0.54	(-1.09, 0.02)	-0.53	(-1.09, 0.02)
**Present smoke, no snuff**	447	4.33	(3.97, 4.68)	-0.23	(-0.66, 0.21)	-0.17	(-0.62, 0.27)	-0.14	(-0.59, 0.31)
**Present use of both smoke and snuff**	21	3.51	(1.86, 5.15)	-1.03	(-2.68, -0.63)	-0.99	(-2.65, 0.67)	-0.93	(-2.60, 0.73)

Model 1: adjusted for age; Model 2: adjusted for age, education and income; Model 3: adjusted for age, education, income and PAI; N: number of participants; FMD: Flow mediated dilation, %; 95% CI: 95% confidence interval; PAI: Physical activity index, Ref.; Reference group

**Table 3:** Flow mediated dilation according to tobacco use and physical activity.

		N	Unadjusted	Model 1		Model 2	
Recommended PAL				Diff.	95% CI	Diff.	95% CI
**No smoke or snuff**	Yes	258	4.68	Ref.	Ref.	Ref.	Ref.
	No	621	4.45	-0.17	-0.72, 0.39	-0.16	-0.73, 0.40
**Smokers**	Yes	87	4.23	-0.56	-1.49, 0.37	-0.41	-1.35, 0.53
	No	379	4.30	-0.33	-0.94, 0.28	-0.29	-0.91, 0.33
**Snuff users**	Yes	82	4.52	-0.35	-1.31, 0.61	-0.29	-1.25, 0.68
	No	156	3.90	-0.85	-1.61, -0.08	-0.83	-1.59, -0.06

Model 1: adjusted for age; Model 2: adjusted for age, education and income; N: number of participants; FMD: Flow mediated dilation, %; 95% CI: 95% confidence interval, Ref.; Reference group, PAL: Physical activity level

**Table 4:** Flow mediated dilation in men according to self-reported use of tobacco and peak oxygen uptake.

			N	Unadjusted	Model 1		Model 2	
Aerobic capacity			N	mean	Diff.	95% CI	Diff.	95% CI
**No smoke or snuff**	l	High	488	4.57	Ref.	-	Ref.	-
		Low	349	4.57	0.02	-0.50, 0.55	0.03	-0.50, 0.55
**Smokers**		High	148	4.46	-0.16	-0.85, 0.54	-0.14	-0.84, 0.56
		Low	283	4.32	-0.26	-0.81, 0.30	-0.18	-0.75, 0.39
**Snuff users**		High	120	4.53	-0.19	-0.95, 0.57	-0.19	-0.96, 0.57
		Low	105	3.86	-0.81	-1.61, -0.003	-0.74	-1.55, 0.07

Model 1: adjusted for age; Model 2: adjusted for age, education and income; N: number of participants; FMD: Flow mediated dilation, %; 95% CI: 95% confidence interval, Ref.; Reference group

The lowest FMD (3.51%, 1.86, 5.15) was observed among the few men (n = 21) who used both smoking tobacco and snuff.

There were no difference in FMD between fit non- tobacco users (reference group) versus low fit non-tobacco users (0.03%, -0.50, 0.55, Table 4), or those within recommended (active) versus below recommended (inactive) level of physical activity (-0.16%, 0.73, 0.40, Table 3).

## Discussion

Few studies have examined the association between snuff-use and endothelial function, which is an early marker of cardiovascular risk that may emerge several years prior to clinical signs of disease.

Our data confirm the characteristics of the typical snuff user: younger, more fit, higher education, higher income and a more healthy diet compared to smokers [8], i.e. similar to non-users. Despite this FMD was lower in snuff users than in non-users and similar to smokers; and this trend was consistent even after adjustment for age and socio-economic status. The same trend revealed that unfit snuff users had even lower FMD compared to either smokers or non-users.

Our findings concur with smaller studies in young and healthy subjects, indicating an association between snuff use and impaired endothelial function [65,66]. Our study measured FMD in 1 592 participants after a period of at least 4 hours of fasting, while one of few and smaller studies on endothelial function measured as FMD were performed in 20 healthy middle-aged snuff users (in which ten did a cross-over)[24]; FMD measured 20 and 35 minutes after the administration of 1 gram snuff or placebo showed impaired FMD after snuff-administration. In the other a cross-sectional study of FMD was performed on seventeen apparently healthy volunteers [66] measuring FMD in three groups: 1; smoking for more than 1 year and at least 10 cigarettes/day, 2; using at least two containers of snuff/week, or 3; not using any tobacco product. In that study, brachial artery FMD was significantly impaired in snuff-users and cigarette smokers compared with non-users. Thus, snuff seems to have a negative acute effect and a negative chronic effect on FMD.

Previous studies are not conclusive regarding the association between snuff use and future risk of cardiovascular disease, though some studies indicate an association [67,68]. For example, the Atherosclerosis Risk in Communities study found a 1.3 fold higher incidence of cardiovascular disease in oral tobacco users compared to non-users [69]. In contrast, another study comparing previous snuff-use in men with myocardial infarction to men without myocardial infarction found no increased risk of MI in snuff-users. However, a limitation of that study is that it did not match the control subjects for age or other confounders [70].

A study from 1992 suggested that effects of snuff depend on the user’s age and level of physical activity [71]. In our study, lower age in snuff-users had no positive association with FMD unless combined with high aerobic capacity or high physical activity level. This was an unexpected finding because of the known inverse association between age and FMD [19]. Exercise is known to improve endothelial function in a variety of inflammatory conditions known to cause endothelial dysfunction [72,73], including aging [74]. Independent on modality, exercise enhances nitric oxide bioavailability, thereby improving endothelial function [75]. Exercise reduces blood pressure and decreases resting heart rate. It also alters muscle sympathetic nerve activity [76], thereby blunting the stress response triggered by snuff [77]. Regular physical activity diminishes some of these negative effects, irrespective of whether they result from autonomic effects [25,78,79] or from direct effects on endothelial cells [65,80].

The interaction test for physical activity with tobacco groups was not significant in our study; nevertheless, there was a difference between FMD in snuff users with high versus low aerobic capacity (Table 4) and a trend between tobacco groups (Table 2). The observation that the lowest FMD occurred in men who used both snuff and smoking tobacco, increases suspicion that snuff adds a negative effect on FMD even in smokers (Table 2).

We have previously shown a significant association between systolic blood pressure and endothelial function [61]. Snuff use is associated with increased heart rate and blood pressure [77,81] and with decreased diastolic function [82]. Over time, increased blood pressure and decreased diastolic heart function could explain an association between snuff use and future heart failure [78]. Low FMD may therefore be an early warning-sign, heralding risk of future cardiovascular disease and indicating that inactive snuff-using men might be at an increased risk.

## Strengths and Limitations

The strength of the present study is a large sample size including 1 592 men over a wide age range and directly measured flow mediated dilation. All participants were apparently healthy, without medications and disease conditions known to influence endothelial function. Still, we detected reduced endothelial function in snuff users, especially in physically inactive participants. The cross-sectional design did not allow for conclusions about causality in the associations between tobacco use and endothelial function. Tobacco use and physical activity status were self-reported, possibly involving a risk of misclassification that may have confounded the observed associations.

The self-selection of participants from the healthiest part of the population together with the exclusion of all participants with established cardiovascular disease may have concealed the effects of tobacco use, both snuff and smoking tobacco.

## Conclusions

In our study snuff-users had a clear tendency towards lower endothelial function compared to non-users, and were no better than in smokers, despite younger age and a more favourable cardiovascular risk profile. Inactive snuff-using men had lower endothelial function than their physically active counterparts, indicating that physical activity and cardiorespiratory fitness modified the effect of snuff on endothelial function. Longitudinal studies are needed to further explore the role of reduced endothelial function as a link between snuff use and future cardiovascular disease.

## References

[1] Laytragoon-Lewin N, Bahram F, Rutqvist LE, Turesson I, Lewin F Direct effects of pure nicotine, cigarette smoke extract, Swedish-type smokeless tobacco (Snus) extract and ethanol on human normal endothelial cells and fibroblasts. Anticancer Res 31: 1527–1534. 21617206

[2] Erhardt L (2009) Cigarette smoking: an undertreated risk factor for cardiovascular disease. Atherosclerosis 205: 23–32. 10.1016/j.atherosclerosis.2009.01.007 19217623

[3] Unverdorben M, von Holt K, Winkelmann BR (2009) Smoking and atherosclerotic cardiovascular disease: part II: role of cigarette smoking in cardiovascular disease development. Biomark Med 3: 617–653. 10.2217/bmm.09.51 20477529

[4] Wannamethee SG, Lowe GD, Shaper AG, Rumley A, Lennon L, Whincup P.H. et al (2005) Associations between cigarette smoking, pipe/cigar smoking, and smoking cessation, and haemostatic and inflammatory markers for cardiovascular disease. Eur Heart J 26: 1765–1773. 15817606 10.1093/eurheartj/ehi183

[5] Jenum AK, Graff-Iversen S, Selmer R, Sogaard AJ (2007) [Risk factors for cardiovascular disease and diabetes through three decades]. Tidsskr Nor Laegeforen 127: 2532–2536. 17925822

[6] Solbraa AK, Holme IM, Graff-Iversen S, Resaland GK, Aadland E, et al (2014) Physical activity and cardiovascular risk factors in a 40-to 42-year-old rural Norwegian population from 1975–2010: repeated cross-sectional surveys. BMC Public Health 14. 10.1186/1471-2458-14-569 PMC4064500 24906521

[7] Norway S (2015) Smoking habits, 2014 Statistics Norway pp. Tobacco habits in Norway

[8] Haglund B, Eliasson M, Stenbeck M, Rosen M (2007) Is moist snuff use associated with excess risk of IHD or stroke? A longitudinal follow-up of snuff users in Sweden. Scandinavian Journal of Public Health 35: 618–622. 17852996 10.1080/14034940701436949

[9] Laclaustra M, Frangi AF, Frangi AG, Casasnovas JA, Cia P (2008) Association of endothelial function and vascular data with LDL-c and HDL-c in a homogeneous population of middle-aged, healthy military men: Evidence for a critical role of optimal lipid levels. Int J Cardiol 125: 376–382. 17477994 10.1016/j.ijcard.2007.03.001

[10] Kallio K, Jokinen E, Raitakari OT, Hamalainen M, Siltala M, Volanen I, et al (2007) Tobacco smoke exposure is associated with attenuated endothelial function in 11-year-old healthy children. Circulation 115: 3205–3212. 17548727 10.1161/CIRCULATIONAHA.106.674804

[11] Celermajer DS, Sorensen KE, Gooch VM, Spiegelhalter DJ, Miller OI, Sullivan ID, et al (1992) Non-invasive detection of endothelial dysfunction in children and adults at risk of atherosclerosis. Lancet 340: 1111–1115. 1359209 10.1016/0140-6736(92)93147-f

[12] Kuvin JT, Patel AR, Sliney KA, Pandian NG, Rand WM, Udelson JE, et al (2001) Peripheral vascular endothelial function testing as a noninvasive indicator of coronary artery disease. J Am Coll Cardiol 38: 1843–1849. 11738283 10.1016/s0735-1097(01)01657-6

[13] Gokce N, Keaney JF Jr., Hunter LM, Watkins MT, Nedeljkovic ZS, Menzoian JO, et al (2003) Predictive value of noninvasively determined endothelial dysfunction for long-term cardiovascular events in patients with peripheral vascular disease. J Am Coll Cardiol 41: 1769–1775. 12767663 10.1016/s0735-1097(03)00333-4

[14] Asselbergs Folkert W.a b, van der Harst T, Pima,b, Jessurun Gillian A.J.b, Tio Rene´ A.b WHvG, b (2005) Clinical impact of vasomotor function assessment and the role of ACE-inhibitors and statins. Vascular Pharmacology 42: 125–140. 15792930 10.1016/j.vph.2005.01.009

[15] Anderson TJ, Meredith IT, Yeung AC, Frei B, Selwyn AP, Ganz P, et al (1995) The effect of cholesterol-lowering and antioxidant therapy on endothelium-dependent coronary vasomotion. N Engl J Med 332: 488–493. 7830729 10.1056/NEJM199502233320802

[16] Gokce N, Keaney JF Jr., Hunter LM, Watkins MT, Menzoian JO, Vita JA, et al (2002) Risk stratification for postoperative cardiovascular events via noninvasive assessment of endothelial function: a prospective study. Circulation 105: 1567–1572. 11927524 10.1161/01.cir.0000012543.55874.47

[17] Golbidi S, Laher I (2013) Exercise and the Aging Endothelium. Journal of Diabetes Research. 10.1155/2013/789607 PMC3747387 23984434

[18] Bolduc V, Thorin-Trescases N, Thorin E (2013) Endothelium-dependent control of cerebrovascular functions through age: exercise for healthy cerebrovascular aging. Am J Physiol Heart Circ Physiol 305: H620–633. 10.1152/ajpheart.00624.2012 23792680

[19] Skaug EA, Aspenes ST, Oldervoll L, Morkedal B, Vatten L, Wisloff U, et al (2013) Age and gender differences of endothelial function in 4739 healthy adults: the HUNT3 Fitness Study. Eur J Prev Cardiol 20: 531–540. 10.1177/2047487312444234 22456692

[20] Zeiher AM, Schachinger V, Minners J (1995) Long-term cigarette smoking impairs endothelium-dependent coronary arterial vasodilator function. Circulation 92: 1094–1100. 7648652 10.1161/01.cir.92.5.1094

[21] Guarino F, Cantarella G, Caruso M, Russo C, Mancuso S, Aridiacono G, et al Endothelial activation and injury by cigarette smoke exposure. J Biol Regul Homeost Agents 25: 259–268. 21880215

[22] Granberry MC, Smith ES 3rd, Troillett RD, Eidt JF (2003) Forearm endothelial response in smokeless tobacco users compared with cigarette smokers and nonusers of tobacco. Pharmacotherapy 23: 974–978. 12921243 10.1592/phco.23.8.974.32871

[23] Suzuki H, Gao XP, Olopade CO, Jaffe HA, Pakhlevaniants S, Rubinstein I, et al (1996) Aqueous smokeless tobacco extract impairs endothelium-dependent vasodilation in the oral mucosa. J Appl Physiol 81: 225–231. 8828668 10.1152/jappl.1996.81.1.225

[24] Rohani M, Agewall S (2004) Oral snuff impairs endothelial function in healthy snuff users. Journal of Internal Medicine 255: 379–383. 14871462 10.1046/j.1365-2796.2003.01279.x

[25] Fant RV, Henningfield JE, Nelson RA, Pickworth WB (1999) Pharmacokinetics and pharmacodynamics of moist snuff in humans. Tobacco Control 8: 387–392. 10629244 10.1136/tc.8.4.387 PMC1759743

[26] Luo J, Ye W, Zendehdel K, Adami J, Adami HO, Bofetta P, et al (2007) Oral use of Swedish moist snuff (snus) and risk for cancer of the mouth, lung, and pancreas in male construction workers: a retrospective cohort study. Lancet 369: 2015–2020. 17498797 10.1016/S0140-6736(07)60678-3

[27] Wallenfeldt K, Hulthe J, Bokemark L, Wikstrand J, Fagerberg B (2001) Carotid and femoral atherosclerosis, cardiovascular risk factors and C-reactive protein in relation to smokeless tobacco use or smoking in 58-year-old men. J Intern Med 250: 492–501. 11902817 10.1046/j.1365-2796.2001.00917.x

[28] Bolinder G, Noren A, de Faire U, Wahren J (1997) Smokeless tobacco use and atherosclerosis: an ultrasonographic investigation of carotid intima media thickness in healthy middle-aged men. Atherosclerosis 132: 95–103. 9247364 10.1016/s0021-9150(97)00075-0

[29] Yatsuya H, Folsom AR Risk of incident cardiovascular disease among users of smokeless tobacco in the Atherosclerosis Risk in Communities (ARIC) study. Am J Epidemiol 172: 600–605. 10.1093/aje/kwq191 20688904 PMC2950823

[30] Huhtasaari F, Lundberg V, Eliasson M, Janlert U, Asplund K (1999) Smokeless tobacco as a possible risk factor for myocardial infarction: A population-based study in middle-aged men. J Am Coll Cardiol 34: 1784–1790. 10577570 10.1016/s0735-1097(99)00409-x

[31] Hergens MP, Ahlbom A, Andersson T, Pershagen G (2005) Swedish moist snuff and myocardial infarction among men. Epidemiology 16: 12–16. 15613940 10.1097/01.ede.0000147108.92895.ba

[32] Boffetta P, Straif K (2009) Use of smokeless tobacco and risk of myocardial infarction and stroke: systematic review with meta-analysis. BMJ 339: b3060 10.1136/bmj.b3060 19690343 PMC2728803

[33] Hergens MP, Alfredsson L, Bolinder G, Lambe M, Pershagen G, Ye W, et al (2007) Long-term use of Swedish moist snuff and the risk of myocardial infarction amongst men. J Intern Med 262: 351–359. 17697156 10.1111/j.1365-2796.2007.01816.x

[34] Arefalk G, Hergens MP, Ingelsson E, Arnlov J, Michaelsson K, Lind L, et al Smokeless tobacco (snus) and risk of heart failure: results from two Swedish cohorts. Eur J Cardiovasc Prev Rehabil. 10.1177/1741826711420003 21828223

[35] Rohani M, Agewall S (2004) Oral snuff impairs endothelial function in healthy snuff users. J Intern Med 255: 379–383. 14871462 10.1046/j.1365-2796.2003.01279.x

[36] Norberg M, Stenlund H, Lindahl B, Boman K, Weinehall L (2006) Contribution of Swedish moist snuff to the metabolic syndrome: A wolf in sheep's clothing? Scand J Public Health 34: 576–583. 17132590 10.1080/14034940600665143

[37] Teo KK, Ounpuu S, Hawken S, Pandey MR, Valentin V, Hunt d, et al (2006) Tobacco use and risk of myocardial infarction in 52 countries in the INTERHEART study: a case-control study. Lancet 368: 647–658. 16920470 10.1016/S0140-6736(06)69249-0

[38] Wikstrom AK, Stephansson O, Cnattingius S (2010) Tobacco use during pregnancy and preeclampsia risk: effects of cigarette smoking and snuff. Hypertension 55: 1254–1259. 10.1161/HYPERTENSIONAHA.109.147082 20231527

[39] Wikstrom AK, Cnattingius S, Galanti MR, Kieler H, Stephansson O (2010) Effect of Swedish snuff (snus) on preterm birth. BJOG 117: 1005–1010. 10.1111/j.1471-0528.2010.02575.x 20465559

[40] Langhammer A, Krokstad S, Romundstad P, Heggland J, Holmen J (2012) The HUNT study: participation is associated with survival and depends on socioeconomic status, diseases and symptoms. BMC Med Res Methodol 12: 143 10.1186/1471-2288-12-143 22978749 PMC3512497

[41] HUNT research center NUoSaT, NTNU, (2007) Invitasjon til HUNT3 (invitation for participation in HUNT3). pp. Questionnaire Q1 used in health survey HUNT3.

[42] Aspenes S, Nilsen T, Skaug E, Bertheussen G, Ellingsen Ø, Vatten L, et al (2011) Peak oxygen uptake and cardiovascular risk factors in 4631 healthy women and men. Medicine and Science in Sports and Exercise 43: 1465–1473. 10.1249/MSS.0b013e31820ca81c 21228724

[43] HUNT research center NUoSaT, NTNU, (2007–2008) HUNT3 Questionnaire 1 (NT3BLQ1). pp. Questionnaire in HUNT3 sent together with the invitation to everyone aged 20 years or more.

[44] Tyroler HA (1999) The influence of socioeconomic factors on cardiovascular disease risk factor development. Prev Med 29: S36–40. 10641816 10.1006/pmed.1998.0441

[45] Skaug EA, Aspenes ST, Oldervoll L, Morkedal B, Vatten L, Wisloff U, et al (2013) Age and gender differences of endothelial function in 4739 healthy adults: the HUNT3 Fitness Study. Eur J Prev Cardiolog 20: 531–540. 10.1177/2047487312444234 22456692

[46] Krokstad S, Langhammer A, Hveem K, Holmen T, Midthjell K, Stene T, et al (2012) Cohort Profile: The HUNT Study, Norway. Int J Epidemiol. 10.1093/ije/dys095 22879362

[47] ENVIRON International Corporation Arlington V (2010) Review of the Scientific Literature on Snus (Swedish Moist Snuff). Swedish Match, Stockholm, Sweden and Swedish Match North America, Richmond, Virginia.

[48] Helath NIoP (2014) Health risks of Scandinavian snus consumption (English summary).

[49] Corretti MC, Anderson TJ, Benjamin EJ, Celermajer D, Charbonneau F, Creager MA, et al (2002) Guidelines for the ultrasound assessment of endothelial-dependent flow-mediated vasodilation of the brachial artery: a report of the International Brachial Artery Reactivity Task Force. J Am Coll Cardiol 39: 257–265. 11788217 10.1016/s0735-1097(01)01746-6

[50] Peretz A, Leotta DF, Sullivan JH, Trenga CA, Sands FN, Aulet MR, et al (2007) Flow mediated dilation of the brachial artery: an investigation of methods requiring further standardization. BMC Cardiovasc Disord 7: 11 17376239 10.1186/1471-2261-7-11 PMC1847451

[51] O'Brien E, Asmar R, Beilin L, Imai Y, Mancia G, Mengden T, et al (2005) Practice guidelines of the European Society of Hypertension for clinic, ambulatory and self blood pressure measurement. J Hypertens 23: 697–701. 15775768 10.1097/01.hjh.0000163132.84890.c4

[52] Aspenes ST, Nilsen TI, Skaug EA, Bertheussen GF, Ellingsen O, Vatten L, et al (2011) Peak Oxygen Uptake and Cardiovascular Risk Factors in 4631 Healthy Women and Men. Med Sci Sports Exerc 43: 1465–1473. 10.1249/MSS.0b013e31820ca81c 21228724

[53] Aspenes ST, Nauman J, Nilsen TI, Vatten LJ, Wisloff U (2011) Physical activity as a long-term predictor of peak oxygen uptake: the HUNT Study. Med Sci Sports Exerc 43: 1675–1679. 10.1249/MSS.0b013e318216ea50 21364479

[54] Moyna NM, Thompson PD (2004) The effect of physical activity on endothelial function in man. Acta Physiologica Scandinavica 180: 113–123. 14738470 10.1111/j.0001-6772.2003.01253.x

[55] Siasos G, Tousoulis D, Chrysohoou C, Oikonomou E, Zaromitidou M, Gialafos E, et al (2012) The beneficial effect of physical activity on endothelial function in middle-aged and elderly habitants in an area with increased rates of longevity: IKARIA study. Circulation 125: E806–E806.

[56] Marshall IJ, Wang Y, Crichton S, McKevitt C, Rudd AG, Wolfe CDA et al (2015) The effects of socioeconomic status on stroke risk and outcomes. Lancet Neurol 14: 1206–1218. 10.1016/S1474-4422(15)00200-8 26581971

[57] Andersen KK, Steding-Jessen M, Dalton SO, Olsen TS (2014) Socioeconomic position and incidence of ischemic stroke in Denmark 2003–2012. A nationwide hospital-based study. Journal of the American Heart Association 3. 10.1161/JAHA.113.000762 PMC4310360 25030354

[58] Pollitt RA, Rose KM, Kaufman JS (2005) Evaluating the evidence for models of life course socioeconomic factors and cardiovascular outcomes: a systematic review. BMC Public Health 5: 7 15661071 10.1186/1471-2458-5-7 PMC548689

[59] Nordahl H (2014) Social inequality in chronic disease outcomes. Danish Medical Journal 61: B4943 25370965

[60] Conroy RM, Pyorala K, Fitzgerald AP, Sans S, Menotti A, De Backer G, et al (2003) Estimation of ten-year risk of fatal cardiovascular disease in Europe: the SCORE project. Eur Heart J 24: 987–1003. 12788299 10.1016/s0195-668x(03)00114-3

[61] Skaug EA, Madssen E, Aspenes ST, Wisloff U, Ellingsen O (2014) Cardiovascular risk factors have larger impact on endothelial function in self-reported healthy women than men in the HUNT3 Fitness study. PLoS One 9: e101371 10.1371/journal.pone.0101371 24991924 PMC4081583

[62] HUNT research center NUoSaT, NTNU, (2016) Access to HUNT data and samples for research purposes.

[63] Siegel D, Benowitz N, Ernster VL, Grady DG, Hauck WW (1992) Smokeless tobacco, cardiovascular risk factors, and nicotine and cotinine levels in professional baseball players. Am J Public Health 82: 417–421. 1536359 10.2105/ajph.82.3.417 PMC1694380

[64] Organisation WH (2015) Physical Activity and Adults, Recommended levels of physical activity for adults aged 18–64 years. pp. Global Strategy on Diet, Physical Activity & Health.

[65] Rohani M, Agewall S (2004) Oral snuff impairs endothelial function in healthy snuff users. Journal of Internal Medicine 255: 379–383. 14871462 10.1046/j.1365-2796.2003.01279.x

[66] Granberry MC, Smith ES, Troillett RD, Eidt JF (2003) Forearm endothelial response in smokeless tobacco users compared with cigarette smokers and nonusers of tobacco. Pharmacotherapy 23: 974–978. 12921243 10.1592/phco.23.8.974.32871

[67] Critchley JA, Unal B (2004) Is smokeless tobacco a risk factor for coronary heart disease? A systematic review of epidemiological studies. European Journal of Cardiovascular Prevention & Rehabilitation 11: 101–112. 15187813 10.1097/01.hjr.0000114971.39211.d7

[68] Boffetta P, Straif K (2009) Use of smokeless tobacco and risk of myocardial infarction and stroke: systematic review with meta-analysis. British Medical Journal 339. 10.1136/bmj.b3060 PMC2728803 19690343

[69] Yatsuya H, Folsom AR, Investigators A (2010) Risk of Incident Cardiovascular Disease Among Users of Smokeless Tobacco in the Atherosclerosis Risk in Communities (ARIC) Study. American Journal of Epidemiology 172: 600–605. 10.1093/aje/kwq191 20688904 PMC2950823

[70] Huhtasaari F, Asplund K, Lundberg V, Stegmayr B, Wester PO (1992) Tobacco and Myocardial-Infarction—Is Snuff Less Dangerous Than Cigarettes. British Medical Journal 305: 1252–1256. 1477567 10.1136/bmj.305.6864.1252 PMC1883750

[71] Siegel D, Benowitz N, Ernster VL, Grady DG, Hauck WW (1992) Smokeless Tobacco, Cardiovascular Risk-Factors, and Nicotine and Cotinine Levels in Professional Baseball Players. American Journal of Public Health 82: 417–421. 1536359 10.2105/ajph.82.3.417 PMC1694380

[72] Beck EB, Hoellriegel R, Erbs S, Woitek F, Adams V, Blueher M, et al (2010) Regular physical exercise training improves amount of circulating endothelial progenitor cells and coronary endothelial function in pre-diabetic, adipose patients with coronary heart disease. European Heart Journal 31: 783–783.

[73] Hoellriegel R, Beck E, Woitek F, Adams V, Erbs S, Blueher M, et al (2011). Regular physical exercise training partially corrects metabolic alterations and improves peripheral endothelial function in pre-diabetic, adipose patients with coronary artery disease. European Heart Journal 32: 685–685.

[74] Golbidi S, Laher I (2013) Exercise and the aging endothelium. J Diabetes Res 2013: 789607 10.1155/2013/789607 23984434 PMC3747387

[75] Ashor AW, Lara J, Siervo M, Celis-Morales C, Oggioni C, Jakovljevic DG, et al (2014) Exercise Modalities and Endothelial Function: A Systematic Review and Dose-Response Meta-Analysis of Randomized Controlled Trials. Sports Med. 10.1007/s40279-014-0272-9 25281334

[76] Notarius CF, Murai H, Morris BL, Floras JS (2012) Effect of Fitness on Reflex Sympathetic Neurovascular Transduction in Middle-Age Men. Med Sci Sports Exerc 44: 232–237. 10.1249/MSS.0b013e31822a68a5 21701410

[77] Wolk R, Shamsuzzaman AS, Svatikova A, Huyber CM, Huck C, Narkiewicz K, et al (2005) Hemodynamic and autonomic effects of smokeless tobacco in healthy young men. J Am Coll Cardiol 45: 910–914. 15766828 10.1016/j.jacc.2004.11.056

[78] Arefalk G, Hergens MP, Ingelsson E, Arnlov J, Michaelsson K, Lind L, et al (2012) Smokeless tobacco (snus) and risk of heart failure: results from two Swedish cohorts. Eur J Prev Cardiol 19: 1120–1127. 10.1177/1741826711420003 21828223

[79] Benowitz NL, Porchet H, Sheiner L, Jacob P 3rd (1988) Nicotine absorption and cardiovascular effects with smokeless tobacco use: comparison with cigarettes and nicotine gum. Clin Pharmacol Ther 44: 23–28. 3391001 10.1038/clpt.1988.107

[80] Furie MB, Raffanello JA, Gergel EI, Lisinski TJ, Horb LD (2000) Extracts of smokeless tobacco induce pro-inflammatory changes in cultured human vascular endothelial cells. Immunopharmacology 47: 13–23. 10708806 10.1016/s0162-3109(99)00181-2

[81] Martin JS, Beck DT, Gurovich AN, Braith RW (2010) The acute effects of smokeless tobacco on central aortic blood pressure and wave reflection characteristics. Exp Biol Med (Maywood) 235: 1263–1268. 20719817 10.1258/ebm.2010.009376 PMC2971534

[82] Sundstrom D, Waldenborg M, Emilsson K (2012) Acute effects on the ventricular function in Swedish snuffers: an echocardiographic study. Clin Physiol Funct Imaging 32: 106–113. 10.1111/j.1475-097X.2011.01062.x 22296630

